# Phage Stability Across Conditions: Ensuring Accurate Use of Viral Surrogates in Antiviral Testing

**DOI:** 10.3390/v18030398

**Published:** 2026-03-23

**Authors:** Sabine Poelzl, Clemens Kittinger

**Affiliations:** Diagnostic & Research Institute for Hygiene, Microbiology and Environmental Medicine, Medical University of Graz, 8010 Graz, Austria; sabine.poelzl@medunigraz.at

**Keywords:** bacteriophages, Phi6, Qbeta, MS2, survival, environmental conditions, ISO 22196:2011

## Abstract

Bacteriophages can serve as practical surrogates for human viruses in laboratory tests. They share key structural characteristics but are non-pathogenic and can be handled under lower biosafety conditions. This facilitates experiments on persistence, disinfection and materials-testing while reducing time, costs and logistical requirements. However, phage survival strongly depends on many factors including environmental conditions, such as temperature and relative humidity (RH). This study investigates the survival of bacteriophages Phi6, Qbeta and MS2 under different incubation conditions including storage on a surface, in liquid and in the fridge. Standardized ISO protocols for antimicrobial surface testing and extensions were applied. All phages demonstrated good survival in liquid, although stability was temperature-dependent. At refrigerator temperatures, phages remained stable for several months with only minor reduction in the initial titer. At 50 °C, the two non-enveloped phages survived for up to one week, whereas the enveloped phage Phi6 was inactivated within one day. On glass surfaces, Phi6 exhibited reduced stability and was detectable only up to one week at 25 °C and >90% RH. Qbeta and MS2 survived from several days at 50 °C to two to seven days at 37 °C, depending on RH, and remained detectable at 25 °C regardless of humidity. These differences highlight the importance of carefully selecting incubation conditions, as these directly affect phage stability. In particular, unsuitable conditions for antimicrobial testing may cause phage inactivation and thus lead to false-positive results. Therefore, it is essential to define the conditions under which each phage produces reliable results.

## 1. Introduction

Bacteriophages were first discovered in 1915 by Frederick William Twort, and in 1917, Felix d’Herelle realized their potential to kill bacteria [1]. He therefore invented the term “bacteriophage” (from the Greek word phagein meaning “to devour”) [2], literally meaning “bacteria eater”, which describes their ability to destroy bacterial cells through a process called lysis [3]. Since then, numerous research projects have been conducted on this topic. It is generally estimated that the total number of phage particles on earth is approximately 10^31^, which is ten times higher than the bacterial population. This makes these organisms the most abundant in the biosphere [4,5,6,7,8,9]. The ocean is the largest reservoir for bacteriophages, containing 4 × 10^30^ particles [9], whereas for the human gut 10^15^ phages and approximately 10^8^–10^10^ phages per gram of stool are estimated [10]. The phages mainly influence bacterial dynamics and diversity in their various existing compartments [11,12,13].

In addition to their natural occurrence, bacteriophages are also beneficial in research and medicine. They can be used not only to treat bacterial infections, but also to stimulate an anti-tumor immune response due to their immunogenic properties or for the expression of phage-based vaccines [14]. In addition to the advantageous properties of phage-based therapeutic options, the utilization of phages in a laboratory setting confers further benefits. In general, for laboratory work, these characteristics translate to high specificity to their bacterial host. If an adequate host is absent, constant growth is not possible, since it is self-limiting [15]. Thus, it is possible to perform laboratory diagnostics for rapid and specific detection of bacterial pathogens [16] or to study specific bacterial–phage interactions [17]. New methods are continuously being developed to improve our understanding of these phage–bacterium dynamics. This is important for the development of phage-based therapies, since cell-to-cell heterogeneity affects drug susceptibility [18]. Moreover, bacteriophages can also be used as model organisms for human viruses in laboratory tests. For instance, bacteriophage Φ6 (enveloped, double-stranded RNA) and bacteriophage MS2 (non-enveloped, single-stranded RNA) have relevant structural similarities to their target human viruses, such as the presence of an envelope and a capsid/genome type. Furthermore, they are non-pathogenic to humans and are routinely classified as BSL-1 organisms. Consequently, experiments involving persistence, disinfection, materials testing and high-throughput screens can be conducted under less stringent biosafety conditions. This substantially reduces logistical burden, time and cost compared with experiments using human pathogenic viruses [19,20,21,22,23,24,25]. However, as described by Ackermann et al., it is important to “Know your phage” [26]. In terms of evaluating the antiviral efficacy of surfaces or substances, the environmental conditions used for incubation (temperature and humidity) must not be disregarded. Otherwise, the researchers risk obtaining false-positive results and incorrectly assessing a surface modification as antiviral, when the phage has actually been inactivated by the selected incubation conditions.

As our laboratory has previously conducted experiments with phages to evaluate surface modifications for their antiviral efficacy, this study was created to test our three most common phages for their survival over time under different conditions, highlighting the phages’ varying survival abilities.

## 2. Materials and Methods

### 2.1. The Bacteriophages

The used bacteriophages in this survival study are listed in Table 1 and were purchased from a collection of the Leibniz Institute DSMZ (German Collection of Microorganisms and Cell Cultures GmbH, Braunschweig, Germany). These phages were selected because their structural composition enables them to serve as model organisms for a broad spectrum of human viruses. MS2 and Qβ are well-established surrogates for enteric RNA viruses, while Phi6 is often used as a surrogate for enveloped human viruses such as influenza or SARS-CoV-2.

Bacteriophage propagation was done according to the manufacturer’s specifications: the bacterial strains were cultivated overnight in lysogenic broth (LB, Carl Roth GmbH + Co., Kg, Karlsruhe, Germany) containing CaCl_2_ (Merck KGaA, Darmstadt, Germany) at 25 °C ± 2 °C (*P. syringae*) or at 36 °C ± 2 °C (*E. coli*) and 110 rpm. The overnight cultures (ONCs) were diluted at 1:50/1:100 into 10 mL fresh LB media and grown to OD_600_ 0.2. To determine the cell density by OD_600_, an UV/VIS spectrophotometer (VWR International GmbH, Vienna, Austria) was used. The cultures were infected with an approximate multiplicity of infection (MOI) of 0.1 and then incubated for 4 h at 36 °C ± 2 °C or 25 °C ± 2 °C and 50 rpm. The suspensions were stored at 4 °C overnight. On the next day, the phage lysates were centrifuged (10,000× *g*, 20 min, 4 °C) and then filtered through a sterile syringe 0.2 µm filter (VWR International GmbH, Austria). The phage suspensions were stored at 4 °C and the titers were determined by plaque assay.

### 2.2. Survival on Surface

In order to evaluate the effect of different incubation conditions on the survival of the three bacteriophages, an inert reference material without antiviral efficacy was necessary for the test setup. Therefore, glass surfaces were purchased in a 50 × 50 mm format (Cloeren Technology GmbH, Wegberg, Germany) and were sterilized with 70% ethanol (Merck KGaA, Darmstadt, Germany). The survival test is based on the ISO 22196:2011 measurement of antibacterial activity on plastics and other non-porous surfaces [27] and additionally on the ISO 21702:2019 measurement of antiviral activity on plastics and other non-porous surfaces [28] and the ISO 18071:2016 fine ceramics—determination of antiviral activity of semiconducting photocatalytic materials under indoor lighting environment—test method using bacteriophage Q-beta [29].

The sterile surfaces were inoculated with 200 µL viral suspensions with an expected viral titer of 1–4 × 10^7^ plaque-forming units (PFU)/mL in 0.2% *v*/*v* Tryptone Soy Broth (TSB, Oxoid Ltd., Basingstoke, UK) and were then covered by a sterilized 40 × 40 mm polyethylene film. Immediately after application, the initial concentration was determined (=0 h). The other specimens were incubated for up to one week (=7 d), with survival tests performed daily to gain a more detailed understanding of survival over time (antimicrobial surface tests are limited to 24 h). Different incubation conditions have been used (25 °C, 37 °C or 50 °C and 35–60% relative humidity (RH) or >90% RH). The conditions were selected according to the above-mentioned ISOs (ISO 22196:2011 tests bacteria at 37 °C and >90% RH; ISO 21702:2019 tests phages at 25 °C and >90% RH). However, the high RH of over 90% does not appear realistic for many room conditions, so a lower RH of 30–65% was also included. Additionally, 50 °C was included to cover a higher temperature. After incubation, any remaining infectious viral particles were recovered by using a 10 mL SCDLP medium as a neutralizer containing TSB (Oxoid, Wesel, Germany), lecithin (Carl Roth GmbH + Co., Kg, Karlsruhe, Germany) and Tween^®^80 (Amresco Inc., Solon, OH, USA). After washing four times, dilutions were prepared in peptone saline solution (Carl Roth GmbH + Co., Kg, Karlsruhe, Germany). Then, 0.1 mL of the appropriate host and 1 mL of the viral dilution were added to 2 mL of liquid Top-Agar containing peptone, saline, yeast extract, CaCl_2_, and agar (Carl Roth GmbH + Co., Kg, Karlsruhe, Germany). After gentle mixing, the solution was poured onto LB agar (Carl Roth GmbH + Co., Kg, Karlsruhe, Germany) plates containing CaCl_2_. PFUs were counted after incubation for 24 h at 25 °C ± 2 °C (*P. syringae*) or 36 °C ± 2 °C (*E. coli*).

The applied load is defined as the actual number of viral particles applied to the samples in the experiment. Time point 0 h is defined as the test point where the viral suspension is harvested immediately after pipetting under the foil to ensure initial concentration of each sample. Triplicate determinations (n = 3) were performed for each incubation period to calculate the mean and standard deviation for the survival rate under the various incubation conditions. When no plaques were countable on the plates, the limit of detection was set at 10 PFU, since 10 mL of the neutralization medium was used.

### 2.3. Survival in Liquid

The experiments for liquid testing were based on the setup described above in “Survival on Surface”. Therefore, the same liquid (0.2% *v*/*v* TSB), initial viral concentration (1–4 × 10^7^ PFU/mL) of the same bacteriophages (Phi6, Qbeta and MS2) as well as the same incubation temperatures (25 °C, 37 °C or 50 °C) and incubation period (daily determinations for up to one week) were selected. However, the test setup was adapted from a surface test to a liquid test. An initial suspension of each bacteriophage in 0.2% *v*/*v* TSB was prepared in a 50 mL tube (Greiner Bio-One GmbH, Kremsmünster, Austria). The expected applied load of 1–4 × 10^7^ PFU/mL was determined, before the viral suspension was divided into three (n = 3) 15 mL tubes (Greiner Bio-One GmbH, Kremsmünster, Austria). These tubes were then incubated at either 25 °C, 37 °C or 50 °C for 1 h. Then, aliquots were taken for 1:10 dilutions series and for a plaque assay (see Section 2.2). The remaining viral suspension was again incubated at the same temperature for up to one week (7 d). Aliquots were removed daily to determine the survival of the bacteriophage used by a plaque assay.

The applied load is defined as the actual number of viral particles in liquid before temperature treatment has started. Time point 0 h was not performed in the liquid tests, since this would equate to the applied load. Instead, the incubation time point at 1 h was introduced.

### 2.4. Survival in the Fridge

No separate experiment was conducted for this data; instead, the data was generated automatically over time. In general, the stock solutions of all three bacteriophages which have been used in this study were stored in the fridge at 3 °C to 5 °C in the ONC medium LB + CaCl_2_. Each stock is used until the remaining volume is almost depleted and/or the phage concentration had decreased to a level unsuitable for further experiments. Over the course of storage, multiple experiments or titer determinations were performed. For these assays, appropriate dilutions of the stock suspension were plated on LB agar plates using the corresponding host bacterium (see Section 2.2). These ongoing experiments allowed the collection of data on the decrease in bacteriophage concentrations during long-term storage at refrigerator temperatures over several months. However, the timing and frequency of these determinations, as well as the number of viral stocks tested, were not based on a predefined experimental design. Consequently, the data were generated coincidentally rather than according to a systematic study plan. Data on four (Phi6 and Qbeta) and two (MS2) phage stocks are available over a period of between five and 19 months. The paucity of data on MS2 is explicable, given that this bacteriophage has not been incorporated into the experimental setup for a significant amount of time.

### 2.5. Calculation of the Results

Since the evaluation differs between the ISOs, an equal calculation of the results was chosen to make it comparable. Therefore, the calculation is based on ISO 18071:2016:

N=C∗V∗D

N—the number of still-infectious bacteriophages [PFU];

C—average plate count;

V—volume, in mL, of SCDLP added to the specimen (10 mL);

D—dilution factor for the plates counted.

For “Survival in Liquid” and “Survival in the Fridge”, V was excluded in the calculation, since the SCDLP was not necessary.

The calculation of the reduction compared to the initial concentration was done with the following formula:

$$R=\frac{\left(U_{0}-U_{t}\right)}{U_{0}}\;\mathrm{or}\;R=\frac{U_{0}}{U_{t}}$$

R—decrease in surviving bacteriophages in [%] or [log_10_];

U_0_—the average of the common logarithm of the number of still-infectious viral particles recovered from the samples immediately after inoculation (0 h for “Survival on Surface” and applied load for “Survival in Liquid” and initial titer determination for “Survival in the Fridge”);

U_t_—the average of the common logarithm of still-infectious viral particles recovered from the samples after the different time points.

The verification of the methodology was calculated according to the guideline of ISO 22196:2011 through the 0 h triplicates:

$$(L_{max}-L_{min})L_{mean}\leq 0.2$$

Lmax—log_10_ of the maximum number of still-infectious phage particles found on a specimen;

Lmin—log_10_ of the minimum number of still-infectious phage particles found on a specimen;

Lmean—log_10_ of the mean number of still-infectious phage particles found on the specimens.

A value ≤ 0.2 indicates a valid test result; only calculatable for “Survival on Surface”, since 0 h data is only available here.

### 2.6. Data Analysis

Results were expressed as described with corresponding mean ± standard deviation (SD). Depictions were generated using CorelDRAW 2019 (Corel Coperation, Ottawa, ON, Canada) and GraphPad Prism Version 10 (GraphPad Software, Boston, MA, USA).

## 3. Results

### 3.1. Survival on Surface

**Figure 1:**
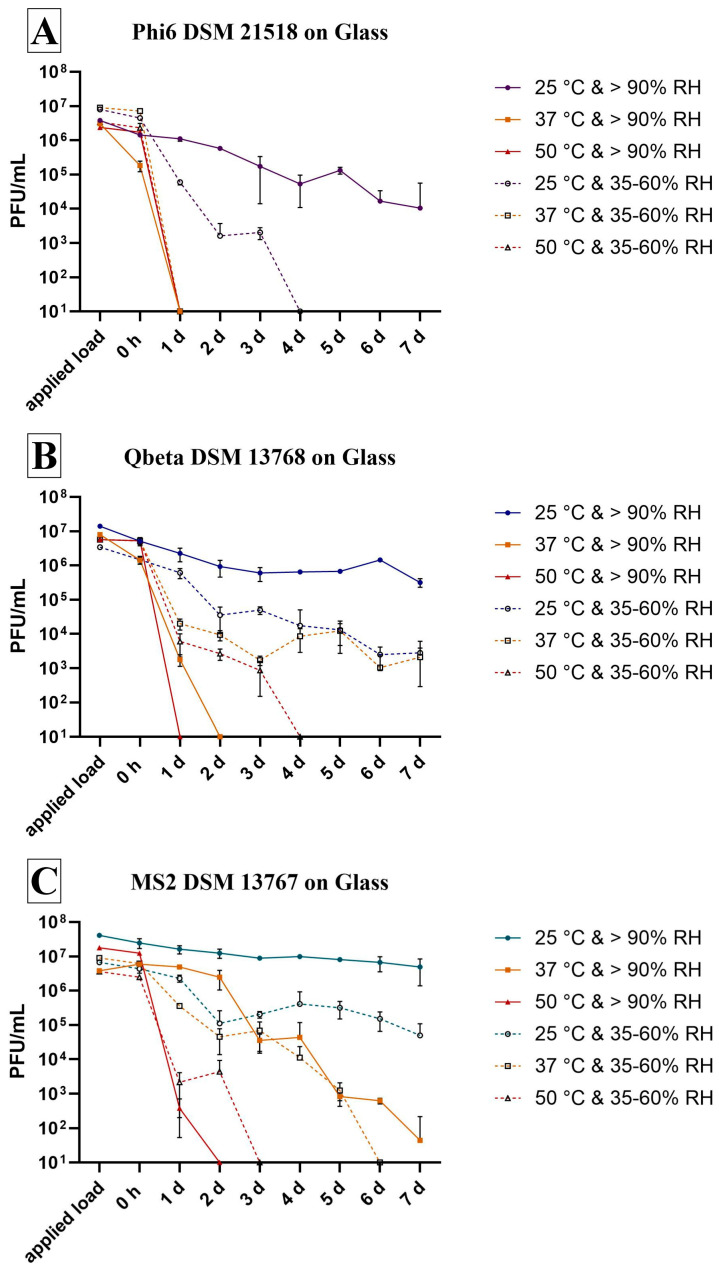
Survival of three different bacteriophages on glass surfaces under different incubation conditions. The bacteriophages Phi6 (**A**), Qbeta (**B**) and MS2 (**C**) were used with an initial concentration of 1–4 × 10^7^ PFU/mL. Incubation on glass was performed at either 25 °C, 37 °C or 50 °C with a relative humidity (RH) of either >90% or 35–60%. The survival of the bacteriophages was monitored daily until no further plaques could be counted or until the seventh day of incubation. Further 0 h shows the recovery immediately after inoculation on glass. The limit of detection was set at 10 PFU. The error bars indicate the standard errors of the respective means, which were calculated from triplicate samples (n = 3).

Glass is recognized as an inert reference material that should have no effect on microorganisms. It was therefore used to determine the survival rate of the bacteriophages under different environmental conditions on a surface for one week. The enveloped bacteriophage Phi6 generally exhibited the lowest persistence (Figure 1A). Over the test period of one week, it could only survive at 25 °C and >90% RH and even then, a decrease of over 2 log_10_ was detectable (Table 2). Incubation at the same temperature (25 °C) and a lower RH (35–60%) resulted in the inactivation of this phage after 4 d. Under the other incubation conditions, rapid inactivation occurred after just 1 d (reduction of over 4 log_10_ or over 5 log_10_, Table 2). The two non-enveloped phages survived well at 25 °C and >90% RH with a smaller decrease compared to Phi6. Qbeta showed a reduction of approximately 1 log_10_ after 7 d, while MS2 decreased by only half as much, showing a reduction of 0.5 log_10_. Furthermore, Qbeta was able to survive the entire test period at both 25 °C and 37 °C with a RH of 35–60% (Figure 1B). Decreases from the initial concentration (0 h) were calculated as 2.5 log_10_ (25 °C) and 3.3 log_10_ (37 °C) (Table 2). At 37 °C with higher RH, complete inactivation occurred after 2 d. At 50 °C, the higher RH of >90% also led to faster elimination (no phages detectable after 1 d), whereas at 35–60% RH, survival could be detected for up to 4 d on glass. In addition to the two RH conditions at 25 °C, MS2 was also able to survive at 37 °C and RH > 90% for up to one week (Figure 1C). However, the survival rate after one week was low at 37 °C and RH > 90%, with approximately 45 plaques. Conditions at 37 °C and 35–60% RH, on the other hand, led to complete inactivation (5.6 log_10_ reduction, Table 2) by the sixth day. Faster elimination after 2 d (>90% RH) or 3 d (35–60% RH) was only achieved by increasing the temperature to 50 °C for MS2.

### 3.2. Survival in Liquid

**Figure 2:**
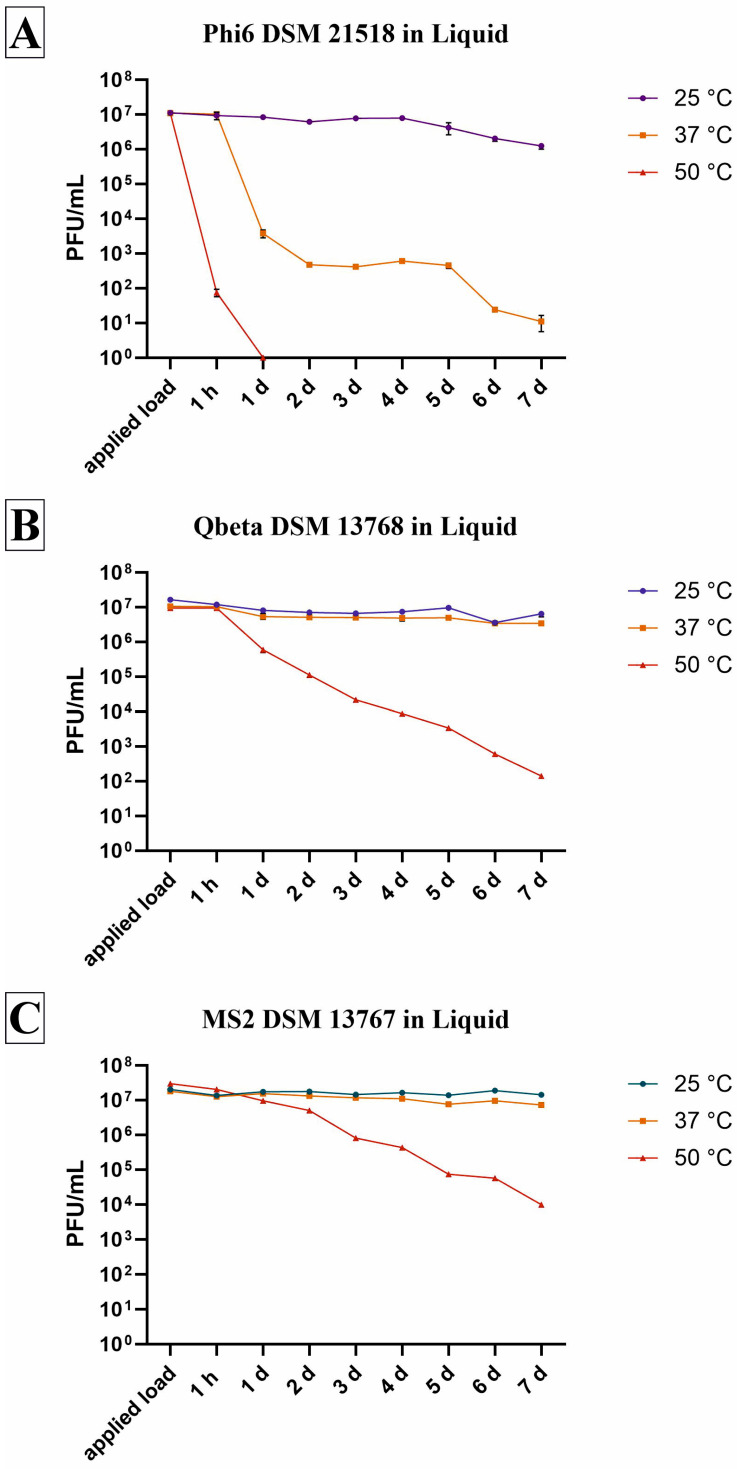
Survival of three bacteriophages in liquid under different incubation temperatures. The bacteriophages Phi (**A**), Qbeta (**B**) and MS2 (**C**) were incubated with an initial concentration of 1–4 × 10^7^ PFU/mL in 0.2% *v*/*v* TSB. Incubation was performed for 1 h to 7 d at either 25 °C, 37 °C or 50 °C. The survival of the bacteriophages was monitored daily until no further plaques could be counted or until the seventh day of incubation. The applied load shows the actual initial concentration before temperature incubation. The error bars indicate the standard errors of the respective means, which were calculated from triplicate samples (n = 3).

The three bacteriophages were incubated in the same liquid as they were on glass and were also exposed to the same temperatures. In liquid incubation, it was also shown that Phi6 exhibited the lowest persistence (Figure 2A), as was already the case on glass surfaces (Figure 1A). The phage exhibited a decline in infectiousness at 25 °C, with a minimal decrease of 0.9 log_10_. In contrast, only 33 plaques were detected in total after a period of 7 d at 37 °C, whereas all phages were eliminated after just one day at 50 °C. Thus, complete reduction by 7.11 log_10_ was achieved at 50 °C in this short amount of time (Table 3). After seven days at 50 °C, Qbeta also showed a high reduction of 4.67 log_10_. However, this non-enveloped Enterobacteria phage demonstrated a high level of resilience at the other two temperatures (Figure 2B). Only a small decrease in infectious phage particles of 0.26 log_10_ at 25 °C and 0.31 log_10_ at 37 °C was calculatable after 7 d (Table 3). MS2 demonstrated the greatest resistance to all temperatures compared to the other phages (Figure 2C). It was observed that at both low temperatures, there was only a minimal loss of infectivity. At a temperature of 25 °C, a reduction of 0.14 log_10_ was observed, while at 37 °C, a reduction of 0.25 log_10_ was recorded in comparison to the initial concentration (Table 3). MS2 also demonstrated a good survival rate at 50 °C, exhibiting a reduction of approximately 3 log_10_ after one week.

In general, the survival rate of all phages was higher under liquid incubation conditions (Figure 2) than on a surface (Figure 1), where dry conditions prevailed at some point. Furthermore, the results obtained in liquid were more consistent than those on glass surfaces. On surfaces, substantial variations were observed between the triplicates at each time point as well as between the time points, likely attributable to the distinct drying times of the medium.

### 3.3. Survival in the Fridge

**Figure 3:**
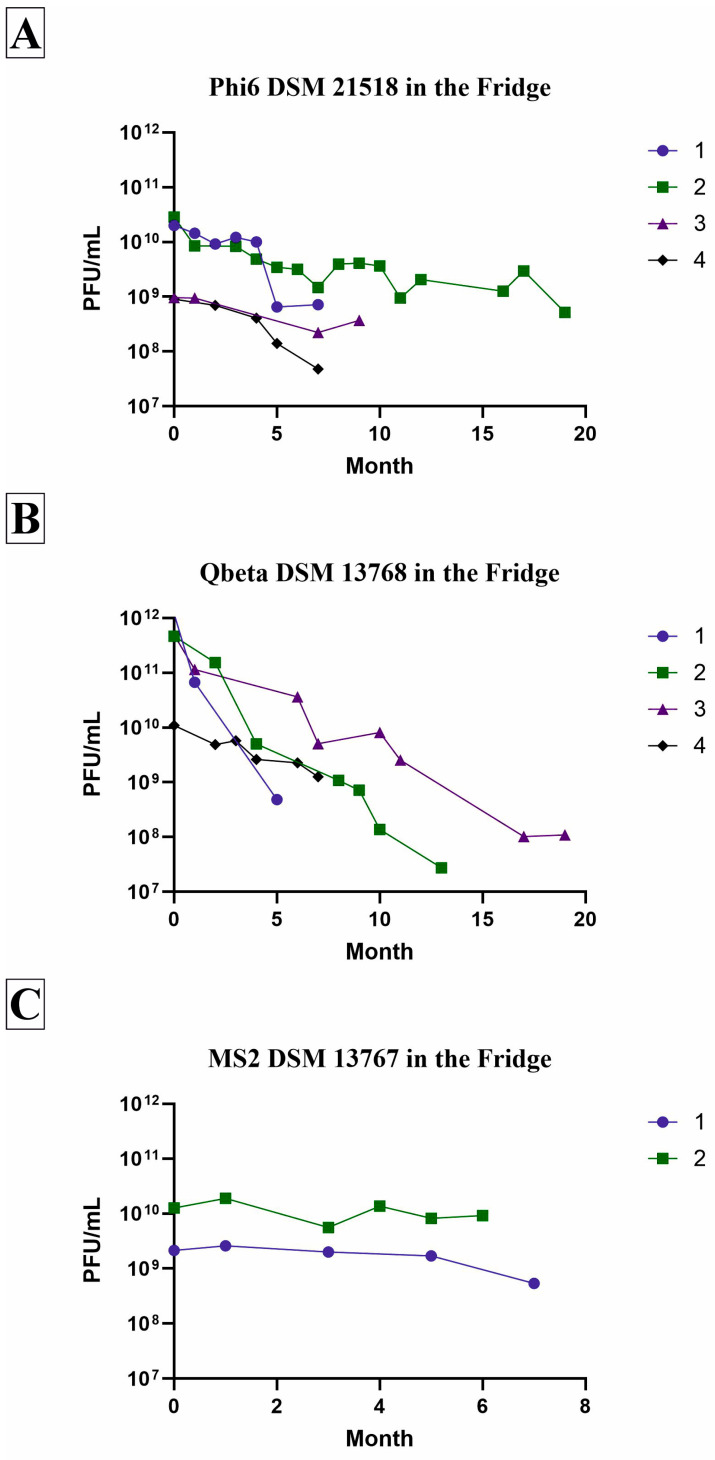
Survival of three different bacteriophages in the fridge over several months. The bacteriophages Phi6 (**A**), Qbeta (**B**) and MS2 (**C**) were stored in the fridge at temperatures between 3 °C and 5 °C for several months. The concentration of the stock solution is thereby determined directly after the propagation of the reproduced phages and frequently due to experiments performed. The measurement points shown may therefore consist of one or more values and are obtained ad hoc. A new phage stock is produced when the concentration reaches a critical point (usually 10^7^ PFU/mL) or when the existing quantity is used up. As MS2 has not been incorporated into the experimental setup for a significant amount of time, only two phage stocks (stock number 1 and 2) have been observed for a shorter period (up to a maximum of seven months). For Phi6 and Qbeta, however, long-term tests of up to 19 months are available for four phage stocks (stock number 1, 2, 3 and 4) each.

The survival of different stocks of the three bacteriophages was investigated during storage at refrigeration temperatures. Phi6 and Qbeta were observed for a total of four years and four different phage stocks each, shown in Figure 3. Phi6 (Figure 3A) remained stable for a longer period of time in the refrigerator than Qbeta (Figure 3B). After seven months, the decrease compared to the initial concentration was between 0.44 log_10_ and 1.28 log_10_ for Phi6 (Table 4). Even after 19 months of storage, the second stock showed a decrease in only 1.5 log_10_. In contrast, the decrease from the Qbeta stocks showed a high deviation between the stocks after 5 to 8 months, ranging from 0.48 log_10_ to 3.25 log_10_. Finally, after over one year of storage, the decrease was high with 4.17 log_10_ (stock 2) and 3.45 log_10_ (stock 3) (Table 5). However, higher initial concentrations for Qbeta of an average of 5 × 10^11^ PFU/mL can be achieved, meaning the phage stocks can be used for a similar length of time to Phi6 phage stocks until the concentration reaches the critical point of 10^7^ PFU/mL. As the MS2 phage was only incorporated into the test setup for slightly more than a year, surveillance data during storage in the fridge is only available for two stocks. However, based on the small amount of data available, it was determined that the second non-enveloped phage remains very stable (Figure 3C). Compared to Qbeta, the decrease in concentration after 5 and 7 months was less than 0.5 log_10_ (Table 5).

## 4. Discussion

Bacteriophages can be used as surrogates for pathogenic human viruses in experiments involving persistence, disinfection and materials testing under reduced biosafety requirements. Nevertheless, it is imperative to understand the environmental conditions that are appropriate for each bacteriophage in order to avoid false results. As shown in previous studies, glass is an inert reference material that has no antimicrobial effect [30,31,32]. This study therefore used glass to examine the stability of bacteriophages on a surface over a period of one week under different environmental conditions. Stability tests were also performed in liquid at different temperatures. This showed that the bacteriophage MS2 survived for the longest period under most conditions observed, compared to the other bacteriophages (Figure 1, Figure 2 and Figure 3). Other research studies conducted under different conditions have also found that MS2 is often more persistent. For example, compared to Phi6, MS2 survives better in dust, on carpets and in liquid droplets, as well as when disinfectants are employed [33,34,35]. MS2 is a comparatively stable virus due to the absence of an outer lipid envelope, making it more resistant to common disinfectants and extreme conditions [36]. Phi6, on the other side, has a lipid envelope which might be essential for infection of the host bacterium [37], but it makes it more attackable. Higher temperatures of 37 °C and 50 °C in particular had a severe effect on these phages’ viability incubated on glass (Figure 1A) and in liquid (Figure 2A), respectively. Gomes et al. also found that phage survival was significantly lower at 37 °C than at 25 °C or 17 °C. In that study, the viability of phage Phi6 fell to the detection limit of the method after only 24 h at 37 °C, whereas at lower temperatures, the phage remained detectable for weeks [38]. A further study investigated the effectiveness of a phage mixture in biocontrolling a specific strain of *P. syringae*, finding that the viability decreased at 44 °C [39]. This temperature sensitivity can be explained in the context of the host bacterium (*P. syringae*) being a plant pathogen, for which an optimum temperature of 28 °C is expected. At higher temperatures, growth is reduced and virulence mechanisms may be affected [40,41]. It seems that Phi6 has adapted well to the temperatures of its host. Another study investigated the effect of humidity on the infectivity of aerosolised Phi6, showing a characteristic V-shaped curve with a greater decrease in infectivity at 25% and 75% RH [42]. However, the data are not directly comparable with those of this study, since tests involving aerosolised bacteriophages have not been performed. Interestingly, Qbeta also exhibited reduced viability at higher RH and/or temperature on glass (Figure 1B), as well as in the liquid experiments (Figure 2B). Qbeta showed greater resistance only under conditions of 35–60% RH and 37 °C or 50 °C on glass (Figure 1B), as indicated by its lower reduction compared to MS2 (Table 2). The different behavior of the two non-enveloped and single-stranded RNA phages may be due to differences in the molecular weight of their capsid and A proteins, and variations in their protein composition [43].

In addition to the primary study, data on stability at refrigerator temperatures was compiled over a long period of time. This data was collected over four years for Phi6 and Qbeta, and over one year for MS2, revealing only a slight decrease in concentration over time. As demonstrated by Alvi et al., storage at 4 °C is the most effective way to maintain stable concentrations of bacteriophages for up to one year [44]. However, it was also described that the suitable storage temperature depends on the phage type used. For instance, some tailed and filamentous phages without lipids can be stored for 5–10 years and their titers should be expected to decrease 1 log per year [26]. The present study also observed differences (Figure 3). Qbeta demonstrated a more rapid decline in concentration over time compared to the other two phages. However, the MS2 test period was insufficiently prolonged to facilitate precise comparisons between the two non-enveloped bacteriophages.

Another limitation of this study relates to the restriction of the chosen media. The medium used for the ONCs of the bacteria and ongoing the propagation/purification of the phages (LB + CaCl_2_) was also used to store the phages in the fridge. Highly diluted TSB was used for the other experiments (survival on surface and in liquid), since it is the applied medium for ISO 22196:2011 [27]. Both media contain NaCl, which is known to be a stabilizer for the storage of bacteriophages [45]. In other media, especially in the “Survival in Liquid” experiments, the results would probably be different. For instance, a study could show that Phi6 can persist with a half-life of 34 h in spring mineral water incubated at 25 °C [46]. However, in our experiment, using liquid 0.2% *v*/*v* TSB at 25 °C, the concentration did not decrease by half over the one-week testing period. It is therefore advisable to consider additional testing with different media in future investigations.

## 5. Conclusions

This study highlights the importance of understanding virus-specific environmental stability when using bacteriophages. Across the tested conditions, MS2 consistently demonstrated the highest persistence on glass surfaces and in liquid, reaffirming previous findings that its non-enveloped structure confers greater resistance to environmental stressors. In contrast, the enveloped phage Phi6 showed marked sensitivity to higher temperatures, reflecting its adaptation to the lower optimal growth temperature of its plant-associated host. Qbeta exhibited an intermediate stability profile, with notable susceptibility to higher temperatures and relative humidity. It is therefore necessary to ascertain the appropriate conditions to prevent unwanted loss of phage infectivity, based on the selected test setup. This applies to the selected temperature, RH and media, to ensure that no incorrect assessment is made regarding disinfectants or antiviral surface modifications, for example. Consequently, preliminary tests should be conducted to establish suitable negative control with all conditions for the selected phages. Tests involving other conditions, such as excessively high temperatures for Phi6, should be avoided to prevent an inaccurate assessment of the product.

## Figures and Tables

**Table 1:** Bacteriophages and their bacterial hosts used in this study.

Bacteriophage	DSMZ	Bacterial Host	DSMZ
Phi6 (ϕ6)	DSM 21518	*Pseudomonas syringae* (*P. syringae*)	DSM 21482
Qbeta (Qβ)	DSM 13768	*Escherichia coli* (*E. coli*)	DSM 5210
MS2	DSM 13767	*Escherichia coli* (*E. coli*)	DSM 5210

**Table 2:** Reduction in the viral start concentration on glass surfaces under different incubation conditions from one to the seventh day and test validity. The decrease after every day of incubation was calculated in percentage or log_10_ by the results of the initial concentration (=0 h) for every bacteriophage. * refers to those results where no more reduction was calculatable (i.e., at the previous day, no phages were detectable). For the results of the test validity, the values (n = 3) of 0 h were considered.

Incubation Condition	Time [Days]	Phi6 DSM 21518			Qbeta DSM 13768			MS2 DSM 13767		
		%	log_10_	Test Validity	%	log_10_	Test Validity	%	log_10_	Test Validity
**25 °C** **>90% RH**	1 d	22.38	0.13	0.01	56.36	0.23	0.04	35.04	0.15	0.04
	2 d	58.78	0.24		81.82	0.55		49.73	0.20	
	3 d	87.69	0.81		88.24	0.85		64.17	0.28	
	4 d	96.25	1.27		87.38	0.79		60.26	0.25	
	5 d	90.67	1.11		86.94	0.76		67.56	0.31	
	6 d	98.83	1.86		71.69	0.35		73.05	0.37	
	7 d	99.26	2.14		93.80	1.16		80.27	0.51	
**37 °C** **>90% RH**	1 d	99.99	4.18	0.08	99.88	2.81	0.04	16.85	0.12	0.01
	2 d	*	*		>99.99	5.14		58.37	0.24	
	3 d				*	*		99.40	2.17	
	4 d							99.27	2.14	
	5 d							99.99	3.72	
	6 d							99.99	3.96	
	7 d							>99.99	5.13	
**50 °C** **>90% RH**	1 d	>99.99	5.17	0.01	>99.99	5.52	0.03	>99.99	4.33	0
	2 d	*	*		*	*		>99.99	6.12	
	3 d							*	*	
	4 d									
	5 d									
	6 d									
	7 d									
**25 °C** **35–60% RH**	1 d	98.65	1.74	0.01	58.41	0.24	0.04	47.48	0.19	0.04
	2 d	99.96	3.27		97.59	1.42		97.45	1.40	
	3 d	99.95	3.22		96.64	1.30		95.37	1.22	
	4 d	>99.99	5.45		98.82	1.85		90.68	1.11	
	5 d	*	*		99.10	2.11		92.74	1.14	
	6 d				99.83	2.59		96.51	1.29	
	7 d				99.81	2.53		98.87	1.89	
**37 °C** **35–60% RH**	1 d	>99.99	5.72		99.62	2.26		94.27	1.18	
	2 d	*	*		99.82	2.56		99.27	2.14	
	3 d			0.00	99.97	3.31	0.03	98.89	1.90	0.01
	4 d				99.84	2.61		99.82	2.56	
	5 d				99.77	2.43		99.98	3.50	
	6 d				99.98	3.51		>99.99	5.62	
	7 d				99.96	3.25		*	*	
**50 °C** **35–60% RH**	1 d	>99.99	5.23	0.05	99.88	2.86	0.03	99.91	3.12	0.01
	2 d	*	*		99.95	3.20		99.82	2.57	
	3 d				99.98	3.61		>99.99	5.25	
	4 d				>99.99	5.52		*	*	
	5 d				*	*				
	6 d									
	7 d									

**Table 3:** Reduction in the viral start concentration in liquid under different incubation temperatures from one hour to the seventh day. The decrease after 1 h and every following day until the seventh day of incubation was calculated in percentage or log_10_ by the initial concentration (=applied load) for every bacteriophage. * refers to those results where no more reduction was calculatable (i.e., at the previous day, no phages were detectable).

Incubation Condition	Time [Days]	Phi6 DSM 21518		Qbeta DSM 13768		MS2 DSM 13767	
		%	log_10_	%	log_10_	%	log_10_
**25 °C**	1 h	16.37	0.12	27.47	0.14	32.85	0.15
	1 d	25.30	0.13	50.91	0.20	14.96	012
	2 d	45.24	0.18	57.17	0.23	12.85	0.12
	3 d	30.36	0.14	59.80	0.25	29.11	0.14
	4 d	29.07	0.14	54.75	0.22	19.67	0.12
	5 d	62.37	0.27	42.02	0.17	31.87	0.15
	6 d	81.90	0.56	78.18	0.46	7.15	0.11
	7 d	88.93	0.90	61.01	0.26	30.41	0.14
**37 °C**	1 h	7.21	0.11	0.63	0.10	29.26	0.14
	1 d	99.97	3.30	48.57	0.19	13.33	0.12
	2 d	>99.99	4.23	51.11	0.21	25.93	0.14
	3 d	>99.99	4.27	52.06	0.21	35.19	0.15
	4 d	>99.99	4.18	53.33	0.21	38.70	0.16
	5 d	>99.99	4.24	52.38	0.21	57.41	0.24
	6 d	>99.99	5.46	67.30	0.31	46.30	0.19
	7 d	>99.99	6.10	67.30	0.31	59.44	0.25
**50 °C**	1 h	>99.99	5.15	0.70	0.10	32.00	0.15
	1 d	>99.99	7.11	93.68	1.16	68.00	0.31
	2 d	*	*	98.82	1.85	83.11	0.59
	3 d			99.77	2.44	97.29	1.37
	4 d			99.91	3.11	98.57	1.70
	5 d			99.96	3.28	99.75	2.41
	6 d			99.99	4.16	99.81	2.53
	7 d			>99.99	4.67	99.97	3.30

**Table 4:** Reduction in viral titer of four different Phi6 stocks during storage in the fridge. The decrease in the infectious viral concentration over several months was calculated in percentage or log_10_ by the initial concentration after the phage propagation and purification. X refers to months where no titer determination has been performed. The varying duration of the individual phage stocks is related to the production of a new stock that replaces the old one (the concentration or the quantity may have already been too low for further experiments).

	Phi6 DSM 21518							
Time [Month]	Stock 1		Stock 2		Stock 3		Stock 4	
	%	log_10_	%	log_10_	%	log_10_	%	log_10_
**1** **2** **3** **4** **5** **6** **7** **8** **9** **10** **11** **12** **13** **14** **15** **16** **17** **18** **19**	28.09 53.84 39.30 50.06 96.75 X 96.43	0.14 0.22 0.17 0.20 1.31 X 1.28	70.19 X 70.56 82.79 87.86 88.89 94.86 86.10 85.53 87.08 96.67 92.77 X X X 95.54 89.61 X 98.18	0.34 X 0.34 0.58 0.82 0.90 1.20 0.72 0.69 0.77 1.30 1.14 X X X 1.22 0.96 X 1.55	1.96 X X X X X 77.11 X 61.86	0.10 X X X X X 0.44 X 0.26	X 22.78 X 54.72 84.44 X 94.67	X 0.13 X 0.22 0.64 X 1.20

**Table 5:** Reduction in viral titer of the non-enveloped bacteriophage stocks of Qbeta and MS2 during storage in the fridge. The decrease in the infectious viral concentration over several months was calculated in percentage or log_10_ by the initial concentration after the phage propagation and purification. For Qbeta, there are values for four different stocks, whereas for MS2 there were only two available. X refers to months where no titer determination has been performed. * refers to those months where no reduction was calculable (i.e., increase in concentration compared to initial concentration). The varying duration of the individual phage stocks is related to the production of a new stock that replaces the old one (the concentration or the quantity may have already been too low for further experiments).

	Qbeta DSM 13768								MS2 DSM 13767			
Time [Month]	Stock 1		Stock 2		Stock 3		Stock 4		Stock 1		Stock 2	
	%	log_10_	%	log_10_	%	log_10_	%	log_10_	%	log_10_	%	log_10_
**1** **2** **3** **4** **5** **6** **7** **8** **9** **10** **11** **12** **13** **14** **15** **16** **17** **18** **19**	94.35 X X X 99.96	1.18 X X X 3.25	X 67.31 X 98.92 X X X 99.77 99.85 99.97 X X 99.99	X 0.31 X 1.93 X X X 2.43 2.65 3.34 X X 4.17	76.77 X X X X 92.63 98.97 X X 98.36 99.49 X X X X X 99.98 X 99.98	0.43 X X X X 1.14 1.97 X X 1.61 2.19 X X X X X 3.49 X 3.45	X 55.45 47.73 76.18 X 79.32 88.45	X 0.22 0.19 0.42 X 0.48 0.87	* X 6.51 X 20.70 X 74.88	* X 0.11 X 0.13 X 0.40	* X 56.10 * 35.63 27.56	* X 0.23 * 0.16 0.14

## Data Availability

The original contributions presented in the study are included in the article, further inquiries can be directed to the corresponding author/s.
